# Nutritional Status, Abdominal Obesity, and Predictors of BMI Categories Among Community-Dwelling Older Adults in Southern Thailand: A Cross-Sectional Study

**DOI:** 10.3390/ijerph23081025

**Published:** 2026-08-05

**Authors:** Urai Jaraeprapal, Pornchanuch Chumpunuch, Arthit Boonrodchu, Uraiwan Pantong, Ladda Thiamwong

**Affiliations:** 1 School of Nursing, The Excellence Center of Community Health Promotion, Walailak University, 222 Thaiburi, Thasala District, Nakhon Si Thammarat 80160, Thailand; jurai@wu.ac.th (U.J.); arthit.bo@wu.ac.th (A.B.); upantong@gmail.com (U.P.); 2 College of Nursing, University of Central Florida, Orlando, FL 32816, USA; ladda.thiamwong@ucf.edu

**Keywords:** older adults, nutritional status, body mass index, abdominal obesity, social determinants of health, Southern Thailand

## Abstract

**Highlights:**

**Public health relevance—How does this work relate to a public health issue?**

- Rapid population aging in middle-income countries has intensified the double burden of malnutrition, including both undernutrition and overweight/obesity, among community-dwelling older adults.
- Nutritional screening in later life should combine body mass index (BMI) and waist circumference (WC) to detect both general obesity- and abdominal obesity-related cardiometabolic risk.

**Public health significance—Why is this work of significance to public health?**

- This census-based study of 13,458 older adults across 20 sub-districts in Southern Thailand identifies biological, socio-economic, behavioral, clinical, and anthropometric predictors of BMI categories.
- The findings show how nutritional risks in Southern Thailand are shaped by aging, continued employment, physical activity patterns, chronic disease burden, abdominal obesity, and regional food culture contexts.

**Public health implications—What are the key implications or messages for practitioners, policy makers and/or researchers in public health?**

- Primary healthcare and sub-district health services should integrate WC with BMI in routine nutritional screening for older adults.
- Risk-stratified community interventions are needed, including protein-energy support for underweight-prone older adults, and culturally appropriate dietary and physical activity programs for groups at risk of overweight, obesity, and abdominal obesity.

**Abstract:**

Geriatric nutritional problems are increasingly characterized by the co-existence of undernutrition and obesity, yet community-level evidence from Southern Thailand remains limited. Grounded in the social determinants of health (SDH) framework, this cross-sectional study examines the prevalence and independent predictors of BMI categories and abdominal obesity among 13,458 community-dwelling older adults across 20 sub-districts in Southern Thailand. Anthropometric measurements and structured interview data were analyzed using chi-square tests and multinomial logistic regression, with normal weight as the reference category. Overall, 50.3% of participants were overweight or obese, 40.5% had abdominal obesity, and 7.7% were underweight. Advancing age, continued employment, and asthma were independently associated with higher odds of underweight, whereas abdominal obesity was the strongest predictor of elevated BMI categories, increasing the odds of overweight, obesity Class I, and obesity Class II. Female sex, literacy, dyslipidemia, hypertension, and diabetes were also associated with excess BMI categories. Moderate, regular physical activity was a protective factor. These findings support risk-stratified community interventions that integrate BMI with WC screening and address regional occupational, behavioral, clinical, and food culture determinants of nutritional health in older adults.

## 1. Introduction

Obesity among older adults amounts to a significant and growing public health concern globally [1]. Defined by excessive accumulation of body fat that may impair health, obesity is commonly assessed using the body mass index (BMI). In later life, obesity is linked to numerous adverse outcomes, including hypertension, type 2 diabetes mellitus, dyslipidemia, cardiovascular disease, osteoarthritis, sleep disorders, functional decline, and diminished quality of life [2,3,4]. Concurrently, undernutrition and low body weight persist as major issues within older populations [5,6]. Consequently, the nutritional status of older adults should be conceptualized along a continuum from underweight to overweight and obesity, rather than focusing only on obesity. This comprehensive perspective is particularly relevant among aging populations, where both inadequate and excessive nutrition may co-occur and contribute to elevated vulnerability, morbidity, and long-term care needs [5,6].

Thailand is experiencing rapid population aging, which has heightened focus on the health and nutritional status of older adults [7]. As the older population increases, nutritional challenges have become more apparent in both clinical and community contexts. Obesity and abdominal obesity are of particular concern due to their contribution to chronic disease burden, diminished physical functioning, and rising healthcare costs [8,9,10]. Nutritional assessment in older adults is complicated by age-related reductions in skeletal muscle mass and changes in adipose tissue distribution. The body mass index (BMI) reflects overall body size but does not differentiate between fat and muscle mass or indicate fat distribution. Waist circumference provides additional information regarding central adiposity and may identify cardiovascular abnormalities that BMI alone does not capture [11]. Recent studies demonstrate that the BMI, waist circumference, and waist-to-hip ratio are differently associated with mortality and cardiovascular risk in older adults, including those with a normal BMI but central obesity [12,13]. Therefore, BMI and waist circumference should be interpreted together. However, neither measure can diagnose sarcopenia or sarcopenic obesity without evaluating skeletal muscle mass, muscle strength, and physical performance [14,15]. In Southern Thailand, nutritional challenges are uniquely shaped by regional socio-cultural contexts. The region’s dietary culture, known for its intense flavors, high-sodium intake, and the prevalent use of coconut milk in traditional curries, presents specific metabolic risks [16]. Furthermore, Southern Thailand’s socio-economic structure is deeply tied to agricultural sectors, particularly rubber and palm oil plantations. While these occupations involve high physical exertion during the younger years, the transition toward a more sedentary lifestyle in older age without a corresponding adjustment in caloric intake often leads to a significant mismatch in energy balance and a higher risk of abdominal obesity [17].

Nutritional status among older adults is influenced by a range of interrelated demographic and health-related factors. Age and sex are particularly important considerations when interpreting nutritional patterns, as body composition, appetite, and fat distribution differ with age and between sexes. Advancing age increases susceptibility to undernutrition due to physiological decline, loss of muscle mass, and reduced appetite. In contrast, women are more likely to experience overweight or obesity, which may be attributed to sex-specific differences in body composition and fat distribution [18,19]. Social and economic factors, such as education and literacy, also affect nutritional outcomes, although their impacts are not always consistent. In some contexts, higher educational attainment supports healthier behaviors, while in others, it may correspond with lifestyle patterns characterized by lower physical activity and greater energy intake [20,21]. Behavioral factors, including work status and physical activity, also influence outcomes. The World Health Organization recommends that older adults engage in 150–300 min of moderate-intensity, or 75–150 min of vigorous-intensity, aerobic physical activity per week, together with regular muscle-strengthening activities, to preserve functional capacity and reduce chronic disease risk [22]. Sustaining physical activity in older age may protect against overweight and obesity, whereas continued employment or high energy expenditure may increase the risk of being underweight if nutritional intake is inadequate [6,23].

A further consideration in older adults is the limitation of BMI as a sole measure, as it does not differentiate between fat mass and muscle mass. Consequently, BMI may underestimate nutritional and metabolic risk, notably in individuals with sarcopenia or sarcopenic obesity [4,11]. Sarcopenia, defined as the progressive loss of skeletal muscle mass and strength, and its co-occurrence with excess adiposity—termed sarcopenic obesity—have been formally characterized through international consensus criteria [24,25]. An older adult may present with a normal or low BMI while possessing excess abdominal fat and diminished muscle mass. Therefore, WC functions as a valuable complementary indicator in research involving older populations [11,13]. Assessing both BMI categories and abdominal obesity delivers a more comprehensive evaluation of nutritional risk [26,27]. This approach is especially pertinent for community-dwelling older adults, whose health status and daily living circumstances can vary widely. Such a perspective stresses the importance of evaluating nutritional status in relation to both excess body weight and broader age-related changes in body composition [28,29].

Although nutritional problems in older adults are well recognized, community-based evidence from Southern Thailand remains limited, particularly at the local level. Crucially, there is a lack of evidence regarding the specific demographic, socio-economic, and behavioral factors that accurately predict nutritional status in this unique setting. While general determinants have been studied elsewhere, their predictive power within the specific cultural and occupational landscape of Southern Thailand has not been fully established. Local communities differ in demographics, education, occupations, daily activities, and lifestyles, all of which influence nutritional status [16]. In the absence of area-specific data, it is not possible to identify vulnerable groups or map the distribution of nutritional risks. Multi-community studies are required to describe the current situation and to identify factors associated with various BMI categories among older adults in Southern Thailand. Such information can enhance community screening, facilitate early risk identification, and direct targeted health promotion strategies at older adults [30,31].

Accordingly, the current study aims to: (1) assess the nutritional status of community-dwelling older adults across 20 sub-districts in Southern Thailand; (2) determine the prevalence of various BMI categories and abdominal obesity; and (3) identify their key predictors. Grounded in the social determinants of health (SDH) framework [32], our investigation conceptualizes nutritional outcomes as a product of multiple overlapping dimensions. Under this lens, we utilize multinomial logistic regression to examine how multilevel factors spanning biological, demographic, socio-economic, and behavioral determinants influence nutritional status within this unique regional context. Ultimately, by mapping these localized risks, our findings seek to inform tailored nutritional guidelines, community-based screening protocols, and targeted prevention strategies that address the specific sociocultural and occupational realities of the aging population in Southern Thailand.

## 2. Materials and Methods

This study employed a cross-sectional design using baseline data from the first (situational analysis) phase of a larger participatory action research (PAR) project entitled The Systems and Innovation Development for Care of the Elders by Community. This baseline phase was designed as a descriptive, population-based assessment conducted before, and independently of, the subsequent participatory phases involving the co-design, implementation, and evaluation of community-based interventions. The cross-sectional design was deliberately chosen to characterize the nutritional status, abdominal obesity, and factors associated with BMI categories among community-dwelling older adults before any intervention was developed or implemented. The survey was conducted between June and December 2021 in 20 sub-districts across seven provinces in Southern Thailand. Using a census sampling approach, the target population comprised 21,423 older adults residing in the selected communities. Eligible participants included community-dwelling residents aged ≥60 years who had lived in the target area for at least six months, were able to communicate, and provided written informed consent. Individuals with severe cognitive impairment or who were bedridden and unable to undergo anthropometric assessment were excluded. Following the recruitment and eligibility assessment process, 13,872 older adults were included in the initial dataset. Because recruitment was conducted as part of a community-wide census survey coordinated by trained village health volunteers, the numbers excluded according to each specific eligibility criterion and the number who declined participation were not systematically recorded. During data cleaning, 414 participants were excluded because of incomplete data on one or more study variables. A complete-case analysis was performed; therefore, 13,458 participants with complete data were included in the final analysis.

Data were collected using a structured questionnaire comprising: (1) the Demographic and Socioeconomic Factors, (2) Health Behaviors, (3) Clinical Factors, (4) Social and Community Support and (5) Clinical assessments. Information regarding all items was obtained based on a review of the literature. The dietary behavior questionnaire was developed by the research team based on the Thai Health Promotion Foundation handbook developed by Dr. Khanat Kruthkul [33]. The Content validity index was established through review by 3 experts (IOC = 0.75). The pilot was tested with 30 older adults before data collection, and the questionnaire demonstrated acceptable internal consistency (Cronbach’s alpha = 0.8). The dietary behavior questionnaire consisted of 14 items, and each item was rated on a three-point Likert scale ranging from 1 (never) to 2 (sometimes), and 3 (always). Item scores were summed to obtain a total score ranging from 14 to 42. The total score was classified into three levels based on the percentage of the maximum possible score: good (≥80%; 34–42), moderate (60–79%; 25–33), and poor (<60%; 14–24).

The physical activity questionnaire was developed by the research team based on the World Health Organization (WHO) Global Recommendations on Physical Activity for Health [34], asking participants, “How many days per week do you engage in at least 30 min of moderate-intensity physical activity?” Moderate-intensity physical activity was defined as activities that require moderate effort and noticeably increase heart rate and breathing, such as brisk walking, cycling at a regular pace, gardening, dancing, or household chores. The answers were categorized as regular (>3 times/week), moderate (3 times/week), occasional (1–2 times/week) and never. A clinical assessment tool was obtained following standardized procedures using calibrated equipment.

The questionnaires were reviewed by 3 experts on geriatric nursing, community health nursing and research methodology to evaluate content validity using a content validity (IOC) of 0.75. The overall questionnaire was pilot tested with 30 older adults before data collection, achieving a Cronbach’s alpha coefficient of 0.80.

Nutritional status was classified using the WHO Asian BMI criteria, and abdominal obesity was defined by WC using Asian-specific cut-off values (men > 90 cm, and women > 80 cm) [32]. To ensure data reliability, all equipment underwent daily calibration. Data collectors (health professionals and volunteers) participated in a standardized training workshop to minimize inter-rater variability.

The study protocol was approved by the Ethics Review Committee of Research Involving Human Research Subjects, Walailak University (protocol code WUEC-19-054-03, approved 10 June 2021) [35]. Written informed consent was obtained from all participants prior to data collection, ensuring anonymity and the voluntary nature of participation.

Statistical analysis was performed using IBM SPSS Statistics version 25.0. The analysis proceeded in three distinct stages:

1. Descriptive statistics—frequencies and percentages were used to summarize baseline sociodemographic data;
2. Bivariate analysis—chi-square tests were employed to identify factors significantly associated with BMI categories, using a significance threshold of *p* < 0.05 to select variables for the subsequent regression model;
3. Multivariate analysis—multinomial logistic regression was utilized to identify independent risk factors and control for confounding variables.

The dependent variable (BMI) was treated as a nominal variable with five levels, using “normal weight (BMI 18.5–22.9 kg/m^2^)” as the reference category. Initially, ordinal regression was considered; however, the test of parallel lines was rejected (χ^2^ = 276.725, df = 96, *p* < 0.001), necessitating the use of the multinomial model. Model fit was assessed using log likelihood, deviance statistics, and Nagelkerke R^2^. Multicollinearity was assessed before model fitting, and no evidence of problematic multicollinearity was observed (all variance inflation factor [VIF] values were below 2.5). Results are presented as adjusted odds ratios (AORs) with 95% confidence intervals (CIs).

## 3. Results

### 3.1. Baseline Characteristics and Nutritional Status

A total of 13,458 community-dwelling older adults were included in this analysis, of whom 59.3% were female and 40.7% were male. An overview of baseline sociodemographic characteristics and the distribution of nutritional status across BMI categories is presented in Table 1. Based on WHO Asian BMI cut-off criteria, 42.0% of participants were classified as having normal weight (BMI 18.5–22.9 kg/m^2^). The study revealed a double burden of malnutrition: 50.3% of participants were classified as overweight or obese, comprising 21.9% overweight (BMI 23.0–24.9 kg/m^2^), 22.3% obesity Class I (BMI 25.0–29.9 kg/m^2^), and 6.0% obesity Class II (BMI ≥ 30.0 kg/m^2^). Conversely, 7.7% of participants were classified as underweight (BMI < 18.5 kg/m^2^). Among all participants, 40.5% had abdominal obesity as defined by WC exceeding 90 cm in men and 80 cm in women.

### 3.2. Bivariate Analysis: Factors Associated with BMI Categories

Chi-square analysis revealed that most sociodemographic, behavioral, health-related, and social support factors were significantly associated with BMI categories (*p* < 0.05). The factor with the strongest statistical association was abdominal obesity (χ^2^ = 1137.63, *p* < 0.001), followed by age group (χ^2^ = 242.36), literacy status (χ^2^ = 240.32), sex (χ^2^ = 118.52), smoking status (χ^2^ = 117.02), employment status (χ^2^ = 110.07), and alcohol consumption (χ^2^ = 93.45).

Regarding sociodemographic factors, the prevalence of underweight increased progressively with age, from 4.9% in those aged 60–69 years to 12.6% in those aged 80 years and older, while the proportion of overweight or obese participants declined. Female participants exhibited a higher combined prevalence of overweight and obesity (51.7%) than their male counterparts (48.4%), whereas underweight proportions were similar across sexes. With respect to education and literacy, participants with no formal education, or those who were illiterate, had the highest proportions of underweight (11.1% and 13.6%, respectively); in contrast, those with graduate-level education or who were literate showed a higher prevalence of overweight. Employed older adults had a slightly higher proportion of normal weight (43.5%) than non-employed participants (40.6%), but also a higher proportion of underweight (9.6% vs. 5.8%). Income level was the only sociodemographic variable not significantly associated with BMI categories (χ^2^ = 6.68, *p* = 0.154).

Among behavioral and health-related factors, exercise frequency was significantly associated with BMI (χ^2^ = 55.48, *p* < 0.001). Participants who never exercised exhibited the highest proportions of underweight (10.4%) and obesity Class I (25.0%). Both alcohol consumption and smoking status were strongly associated with BMI (*p* < 0.001); specifically, daily drinkers and current smokers had higher proportions of underweight than non-drinkers and non-smokers. Engagement in hobbies and dependence in ADL were also significantly associated with BMI. Dietary behavior showed a statistically significant but modest association with BMI (χ^2^ = 16.90, *p* = 0.031). Among chronic conditions, hypertension, diabetes mellitus, dyslipidemia (all *p* < 0.001), asthma (*p* = 0.001), and heart disease (*p* = 0.022) were significantly associated with BMI categories, whereas knee osteoarthritis, thyroid disease, and musculoskeletal disorders were not.

Abdominal obesity exhibited a statistically significant association with BMI (χ^2^ = 1137.63, *p* < 0.001). Among participants without abdominal obesity, the prevalences of obesity Class I and Class II were 16.0% and 2.7%, respectively. In contrast, among participants with abdominal obesity, 31.1% were classified as obesity Class I and 10.9% as obesity Class II. Correspondingly, underweight was less common among participants with abdominal obesity than among those without abdominal obesity (4.1% vs. 10.1%), whereas the prevalence of normal weight was lower (31.7% vs. 49.5%).

Regarding social support factors, having a caregiver (χ^2^ = 20.71, *p* < 0.001), receiving support from village health volunteers (VHVs; χ^2^ = 29.72, *p* < 0.001), having access to local health services from sub-district organizations (χ^2^ = 46.22, *p* < 0.001), and participating in health promotion activities (χ^2^ = 27.24, *p* < 0.001) were all significantly associated with BMI categories. Participants who received these forms of community support exhibited a higher prevalence of normal weight. In contrast, utilization of physician services, nurse services, and annual health check-ups were not significantly associated with BMI (*p* > 0.05).

### 3.3. Multivariate Analysis: Independent Predictors of BMI Categories

**Figure 1:**
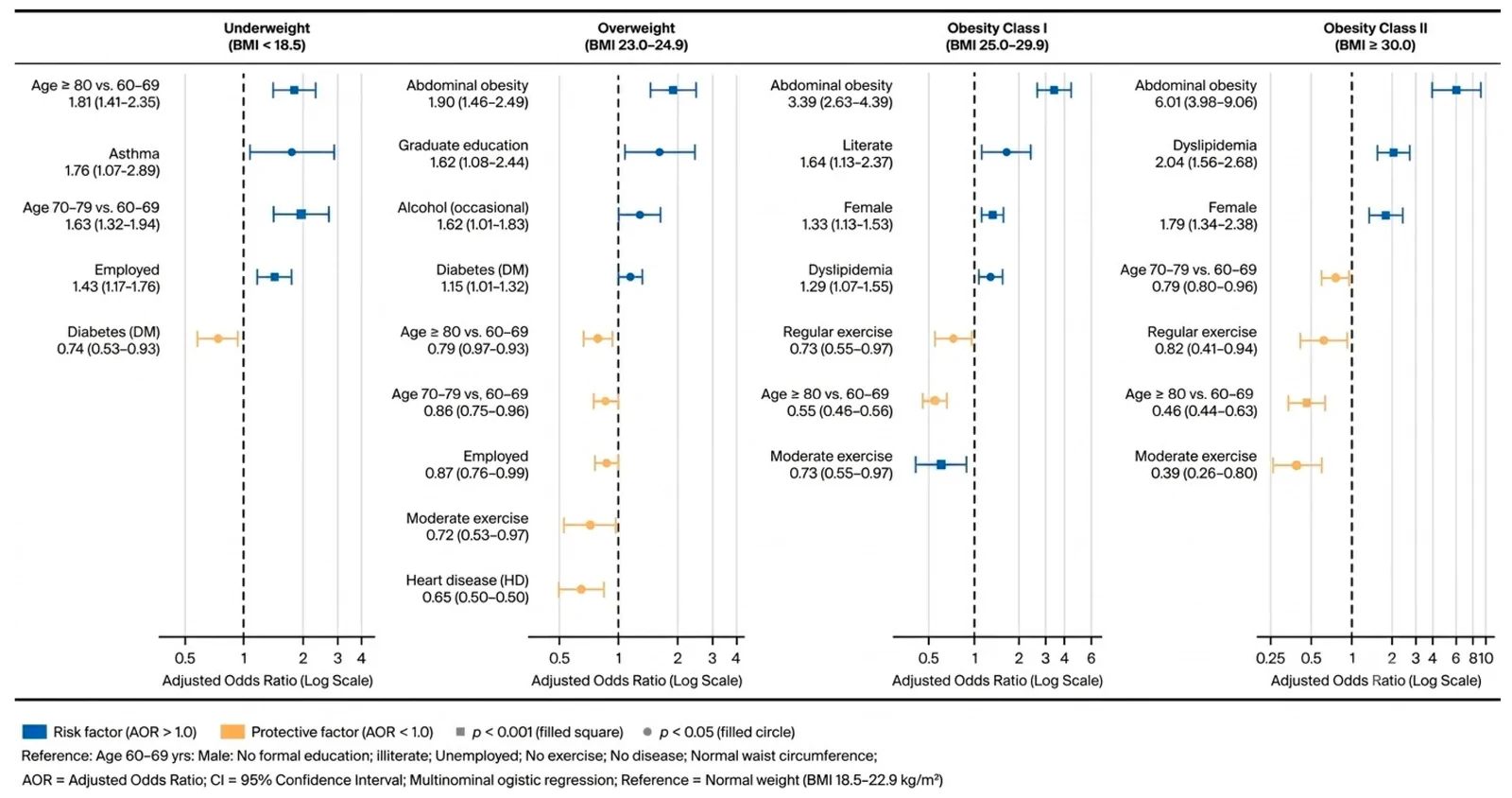
Multivariate analysis of independent predictors of nutritional status (N = 13,458). The dashed vertical line indicates the reference value (AOR = 1.0), and the grey vertical lines represent the logarithmic scale grid lines.

Multinomial logistic regression in Table 2 was conducted to identify independent predictors of each BMI category, using normal weight as the reference. The results are presented as AORs with 95% CI*s* in Figure 1. The overall model fit was acceptable, as indicated by the Nagelkerke R^2^ and deviance statistics. The test of parallel lines was rejected (χ^2^ = 276.73, df = 96, *p* < 0.001), confirming that multinomial logistic regression was the appropriate analytical approach. Multicollinearity was assessed before model fitting, and no evidence of problematic multicollinearity was observed (all variance inflation factor [VIF] values were below 2.5).

#### 3.3.1. Predictors of Underweight

Underweight was defined as a BMI < 18.5 kg/m^2^. Compared with participants aged 60–69 years, those aged 70–79 years (AOR = 1.53, 95% CI: 1.22–1.92, *p* < 0.001) and those aged ≥80 years (AOR = 1.81, 95% CI: 1.41–2.33, *p* < 0.001) had significantly higher odds of underweight. Employed participants also had higher odds of underweight than unemployed participants (AOR = 1.43, 95% CI: 1.17–1.76, *p* = 0.001). Asthma was independently associated with increased odds of underweight (AOR = 1.76, 95% CI: 1.07–2.89, *p* = 0.026). In contrast, diabetes mellitus was associated with lower odds of underweight (AOR = 0.74, 95% CI: 0.58–0.93, *p* = 0.009). Physical activity was not significantly associated with underweight after adjustment.

#### 3.3.2. Predictors of Overweight

Overweight is defined as a BMI of 23.0–24.9 kg/m^2^. Graduate-level education was associated with significantly higher odds of overweight compared to no formal education (AOR = 1.62, 95% CI: 1.08–2.44, *p* = 0.020). Abdominal obesity was also a significant predictor (AOR = 1.90, 95% CI: 1.46–2.48, *p* < 0.001). Conversely, employed participants had lower odds of being overweight than those who were unemployed (AOR = 0.87, 95% CI: 0.76–0.99, *p* = 0.030). Moderate physical activity was independently associated with reduced odds of overweight (AOR = 0.72, 95% CI: 0.53–0.97, *p* = 0.031). Similarly, heart disease was inversely linked to overweight (AOR = 0.65; 95% CI: 0.50–0.84; *p* = 0.001). Conversely, having diabetes mellitus was associated with slightly higher odds of overweight (AOR = 1.15, 95% CI: 1.01–1.32, *p* = 0.042). Furthermore, compared to never-drinkers, occasional alcohol consumption was associated with a modest increase in the odds of being overweight (AOR = 1.29; 95% CI: 1.01–1.63; *p* = 0.039).

#### 3.3.3. Predictors of Obesity Class I

Obesity Class I is defined as a BMI of 25.0–29.9 kg/m^2^. Abdominal obesity emerged as the strongest predictor of Class I obesity (AOR = 3.39, 95% CI: 2.63–4.39, *p* < 0.001). Female sex (AOR = 1.33) and literacy (AOR = 1.64) were also significant risk factors. Additionally, hypertension was associated with modestly higher odds (AOR = 1.17, 95% CI: 1.02–1.34, *p* = 0.028), as was dyslipidemia (AOR = 1.29, 95% CI: 1.07–1.55, *p* = 0.007). Conversely, age ≥ 80 years (AOR = 0.55, 95% CI: 0.46–0.66, *p* < 0.001), employment (AOR = 0.86, 95% CI: 0.76–0.98, *p* = 0.028), moderate physical activity (AOR = 0.55, 95% CI: 0.41–0.73, *p* < 0.001), regular physical activity (AOR = 0.73, 95% CI: 0.55–0.97, *p* = 0.028), and heart disease (AOR = 0.72, 95% CI: 0.55–0.94, *p* = 0.015) were independently associated with lower odds of obesity class I.

#### 3.3.4. Predictors of Obesity Class II

Abdominal obesity was the most potent predictor of Class II obesity, with a six-fold increase in the odds (AOR = 6.01, 95% CI: 3.98–9.06, *p* < 0.001). Dyslipidemia was independently associated with more than a twofold increase in the odds (AOR = 2.04, 95% CI: 1.56–2.68, *p* < 0.001). Female sex was also a significant predictor (AOR = 1.79, 95% CI: 1.34–2.38, *p* < 0.001). Conversely, older age (70–79 years: AOR = 0.76, 95% CI: 0.60–0.96, *p* = 0.020; ≥80 years: AOR = 0.46, 95% CI: 0.34–0.63, *p* < 0.001), moderate physical activity (AOR = 0.39, 95% CI: 0.26–0.60, *p* < 0.001), and regular physical activity (AOR = 0.62, 95% CI: 0.41–0.94, *p* = 0.024) were independently associated with lower odds of obesity class II. Moderate physical activity demonstrated the strongest protective effect, reducing the odds of obesity class II by approximately 61%.

#### 3.3.5. Protective Factor

Dietary behavior, ADL dependency, engagement in hobbies, caregiver availability, VHV support, receipt of local health services, participation in health promotion activities, smoking status, and daily alcohol consumption were not independently associated with any BMI category after adjustment for potential confounders (all *p* > 0.05), despite showing significant associations in the bivariate analysis.

## 4. Discussion

This study examined nutritional status and its associated factors among 13,458 community-dwelling older adults across 20 sub-districts in Southern Thailand. The findings revealed a substantial double burden of malnutrition: 50.3% were overweight or obese, and 7.7% were underweight, consistent with nutritional transition patterns observed in aging populations of middle-income countries [5,6]. Guided by the SDH framework, the results confirm that nutritional outcomes in later life are shaped by intersecting structural, intermediary, and biological determinants operating across multiple levels, including age, sex, education, literacy, employment, physical activity, abdominal obesity, and chronic disease burden.

### 4.1. Strengths

This study has several notable strengths, including its exceptionally large community-based sample (*n* = 13,458), its comprehensive coverage of 20 sub-districts across seven provinces in Southern Thailand, and its use of the social determinants of health (SDH) framework to structure a broad set of structural, intermediary, and biological determinants. The incorporation of cultural and occupational characteristics specific to Southern Thailand adds contextual depth that is rarely represented in the international literature, and the combined assessment of BMI and waist circumference allowed for a more comprehensive evaluation of nutritional risk than either measure alone.

### 4.2. Key Findings and Interpretation

Advancing age was an independent predictor of underweight. Compared to participants aged 60–69 years, those aged 80 years and older had 1.81 times higher odds of underweight (AOR = 1.81, 95% CI: 1.41–2.33), while simultaneously showing lower odds of overweight and obesity, a pattern consistent with the “weight drop in the oldest old” driven by sarcopenia, the anorexia of aging, and progressive physiological decline [6,36,37]. Critically, this inverse relationship between age and BMI-defined obesity should not be equated with reduced nutritional risk, as older adults may simultaneously carry excess abdominal adiposity consistent with sarcopenic obesity, elevating risk for frailty and cardiometabolic complications independently of BMI [11,38].

Female sex was independently associated with higher odds of obesity Class I (AOR = 1.33, 95% CI: 1.13–1.56), and Class II (AOR = 1.79, 95% CI: 1.34–2.38), aligning with evidence from other Asian settings and attributable to postmenopausal adipose redistribution and fat-free mass loss [18,19,39]. Within the SDH framework, these differences may also reflect gendered social roles—including lower occupational physical activity and differential access to leisure-time exercise—that compound biological vulnerability in older Thai women. Notably, visceral adiposity and associated cardiometabolic risk may be underestimated in men with central obesity, but lower overall BMI [40], reinforcing the need for sex-specific screening approaches that assess both general and abdominal adiposity.

Education, literacy, and employment showed context-dependent associations with nutritional status among older adults. Although education and literacy are generally expected to support healthier health behaviors, the current study found that participants with graduate-level education had higher odds of overweight (AOR = 1.62, 95% CI: 1.08–2.44), and literate participants had higher odds of obesity Class I (AOR = 1.64, 95% CI: 1.13–2.37), whereas older adults without formal education were more likely to be underweight. This finding may reflect the influence of the nutrition transition, whereby socioeconomic advancement is accompanied by greater access to energy-dense foods and more sedentary lifestyles [41,42]. In Southern Thailand, this association may be partly explained by regional dietary culture and socio-economic structures. Energy-dense traditional foods, coconut milk-based dishes, sweetened beverages, and local desserts are commonly embedded in daily eating and social gatherings.

Recent evidence from Thailand also indicates that sugar-sweetened beverage consumption varies by socio-economic status and is associated with unhealthy food consumption and low physical activity, supporting the interpretation that more educated or literate older adults may have greater access to obesogenic food environments [43]. In addition, higher educational attainment may be linked to previous administrative or professional occupations, which involve prolonged sitting and lower occupational energy expenditure, while lower educational attainment may reflect lifelong engagement in agricultural or manual work. This may explain why older adults with lower education were less likely to be overweight or obese, but more vulnerable to underweight when dietary intake was insufficient. The bidirectional association of employment further supports this interpretation: employed older adults had higher odds of underweight (AOR = 1.43, 95% CI: 1.17–1.76), but lower odds of overweight and obesity. Employment in this rural context may therefore indicate continued physically demanding work rather than sedentary employment.

Previous evidence also shows that malnutrition among older adults is strongly influenced by socio-economic disadvantage, low income, chronic conditions, and inadequate dietary intake [6,30,44]. Taken together, these findings suggest that education and literacy alone should not be interpreted as universally protective factors against overweight and obesity. Public health interventions in the context of Southern Thailand should adopt a dual strategy: (1) reducing energy-dense food intake, sweetened beverages, and sedentary behavior among more educated or literate older adults, while (2) ensuring adequate energy and protein intake among older adults who remain engaged in manual or agricultural work.

Physical activity was the most influential modifiable correlate identified. Moderate Physical activity was associated with 28% lower odds of overweight (AOR = 0.72), 45% lower odds of obesity Class I (AOR = 0.55), and 61% lower odds of obesity Class II (AOR = 0.39, 95% CI: 0.26–0.60), consistent with meta-analytic evidence linking sustained physical activity to reduced abdominal adiposity and systemic inflammation in older populations [23,45]. The absence of significant associations for occasional physical activity—and the small size of this subgroup (*n* = 25) suggests a possible threshold pattern, whereby only moderate-to-vigorous activity is associated with meaningful metabolic benefit. Public health strategies should therefore consider prioritizing moderate-intensity exercise promotion rather than broadly encouraging any level of activity. These findings suggest that public health interventions should prioritize promoting regular moderate-intensity physical activity, which appears to provide greater metabolic benefits than infrequent activity among older adults.

Dyslipidemia was the strongest disease-related predictor of severe obesity, associated with more than twofold higher odds of Class II obesity (AOR = 2.04, 95% CI: 1.56–2.68), reflecting shared metabolic pathways involving insulin resistance, hepatic lipogenesis, and adipose tissue inflammation [24]. Hypertension was independently associated with obesity Class I (AOR = 1.17, 95% CI: 1.02–1.34), while heart disease showed an inverse association with overweight and obesity—plausibly reflecting disease-related weight loss or dietary modification following diagnosis. Asthma was associated with significantly higher odds of underweight (AOR = 1.76, 95% CI: 1.07–2.89), consistent with evidence that chronic respiratory disease promotes systemic catabolism and reduced appetite [6]. These disease–BMI relationships underscore the importance of integrating nutritional screening within chronic disease management pathways in primary care.

In the present study, 40.5% of community-dwelling older adults had abdominal obesity; in multivariable analysis, abdominal obesity emerged as the strongest independent predictor of elevated BMI, increasing the odds of overweight by 1.90 times, obesity Class I by 3.39 times, and obesity Class II by 6.01 times. These findings align with previous evidence that WC is a sensitive marker of central adiposity and visceral fat accumulation, which is closely linked to insulin resistance, chronic low-grade inflammation, and atherogenic dyslipidemia independently of total body mass [13,26,27,46,47].

Crucially, in aging populations, age-related muscle loss can result in sarcopenic obesity—where individuals present with wasted limbs but protruding abdomens—a dangerous phenotype that is frequently masked by near-normal or modestly elevated BMI [11]. This phenotype reflects the concurrent, age-related loss of skeletal muscle mass and redistribution of adipose tissue toward the abdominal region, such that total body weight—and therefore BMI—may remain largely stable even as body composition shifts unfavorably [24,25]. Consistent with this mechanism, WC emerged as the strongest independent predictor of elevated BMI category in the present study, suggesting that central adiposity, rather than total body mass, more accurately captures the cardiometabolic risk conferred by this age-related shift in body composition. Consistent with this mechanism, waist circumference emerged as the strongest independent predictor of elevated BMI in the present study, increasing the odds of overweight, obesity class I, and obesity class II by 1.90-, 3.39-, and 6.01-fold, respectively. These results strongly support the routine inclusion of WC alongside BMI in community-based nutritional screening protocols for older adults in Thailand to prevent overlooked cardiometabolic risks.

Several community-level social support factors were significantly associated with BMI categories at the bivariate level, including caregiver availability, VHV support (access to local health services and health promotion participation. However, none remained significant independent predictors after adjustment for individual-level determinants. This differs from some studies in home-care populations, where caregiver- and care service-related factors were associated with nutritional outcomes. For example, one study found that caregiver education level was significantly associated with the nutritional status of older adults receiving home care, measured using the mini nutritional assessment [48], while [46] examined older adults receiving home care and highlighted that nutritional status is shaped by functional, health, and care-related factors. These findings suggest that caregiver support and home-care systems may influence nutritional status, particularly when the older adult is dependent or receives structured care [48,49].

The lack of significant associations in the multivariable model may reflect differences in measurement and study context rather than the absence of a true relationship. reflect differences in measurement and context rather than the absence of a true relationship. In the current study, social support variables were measured as dichotomous yes/no indicators, which captured only whether support was present, but not its frequency, duration, intensity, quality, or content. In contrast, previous studies have assessed caregiver characteristics, home-care context, or structured intervention components in greater detail. Evidence from Thailand also shows that VHVs can deliver home visits, personalized counseling, and lifestyle modification support; for example, a VHV-led weight-loss intervention significantly reduced body weight and BMI among adults with obesity, and a VHV-integrated care model improved health behaviors in rural communities [50,51]. Therefore, caregiver support, VHV involvement, and local health services may remain important mechanisms for nutritional care, but future studies should measure support intensity, visit frequency, quality of counseling, and continuity of follow-up to clarify their direct and indirect effects on nutritional status among older adults.

At the policy level, routine screening at sub-district health-promoting hospitals and local community health services should include both BMI and WC to identify underweight, general obesity, abdominal obesity, and possible sarcopenic obesity. Screening results should then be used to classify older adults into priority groups. Older adults aged ≥80 years, those still engaged in physically demanding agricultural work, should be prioritized for undernutrition prevention, while older women, individuals with dyslipidemia, and those with abdominal obesity should be prioritized for obesity and cardiometabolic risk reduction. At the practice level, community nurses, VHVs, caregivers, and local government health services should translate screening results into continuous follow-up rather than one-time assessment. For older adults at risk of underweight, interventions should include home-based dietary assessment, protein-energy intake support, meal planning using locally available foods, and monitoring of food access. For older adults at risk of overweight or obesity, interventions should focus on WC monitoring, culturally appropriate dietary modification, reduction in energy-dense foods, sweetened beverages, and high-fat coconut-milk-based dishes, while preserving the acceptability of Southern Thai food practices. Moderate-intensity physical activity should be promoted through accessible community channels, such as safe walking paths, mosque or temple-based exercise groups, community halls, indoor exercise spaces, and age-friendly group activities. Although caregiver support, VHV involvement, and local health services were not independent predictors in the adjusted model, they remain essential implementation mechanisms for nutritional screening, individualized counseling, referral, and long-term follow-up. Thus, policy should emphasize not only service availability, but also the intensity, quality, continuity, and cultural appropriateness of community-based nutritional care in Southern Thailand.

### 4.3. Limitations

Limitations to the current work include a potential for recall bias: dietary and physical activity behaviors were assessed via self-reported questionnaires, which are susceptible to recall bias and underreporting. Self-reported questionnaires were subject to recall bias. There are limitations inherent in BMI, too: although WC was utilized alongside BMI, BMI alone cannot differentiate between fat mass and muscle mass. In older populations, this limitation may overlook sarcopenic obesity, potentially leading to an underestimation of cardiometabolic risks when relying solely on body weight. According to current international consensus criteria, a definitive diagnosis of sarcopenia or sarcopenic obesity additionally requires objective assessment of muscle strength (e.g., handgrip strength) and muscle mass (e.g., via dual-energy X-ray absorptiometry or bioelectrical impedance analysis) [24,25], none of which were collected in this study. Future research incorporating these measures is warranted to confirm the presence of sarcopenic obesity among older adults with normal or borderline BMI. Our small sub-group sample size was a further limitation. The sub-group of older adults engaging in physical exercise only 1–2 times per week was relatively small (*n* = 25). The sample size restricted statistical power, warranting caution when interpreting and generalizing findings related to this specific cohort.

Additional study limitations include the cross-sectional design. Data were collected at a single point in time. Consequently, temporal relationships and definite causal links between sociodemographic factors, comorbidities, and BMI categories could not be established. The questionnaire asked only “yes” or “no” questions about caregiving, but did not include interview questions about frequency, duration and quality of care. Therefore, we found no impact of predictive factors related to caregivers.

### 4.4. Recommendations for Practice and Future Research

We have several recommendations arising from our study. First, integration of screening tools is recommended for the Southern Thai population, because primary healthcare units should mandate the combined use of WC and BMI in routine health screenings to more accurately identify abdominal obesity among older adults. We also recommend targeted public health interventions. For the oldest adults (aged ≥80 years) and working older adults, interventions should prioritize the prevention of underweight and undernutrition through targeted caloric and nutritional supplementation. For female older adults and individuals with dyslipidemia, weight management and lipid-lowering lifestyle interventions such as intensive weight management programs and lifestyle modification programs should be intensively implemented, as these groups exhibit a significantly higher vulnerability to Class II obesity. Furthermore, we recommend post-screening behavioral modification because our work showed that annual health screening alone did not show a protective effect against high BMI. Local Southern Thai government agencies should plan toward continuous proactive behavioral modification programs focusing on structured, moderate-intensity physical activities tailored to suit older adults.

We also recommend tailored health literacy programs to address the counterintuitive, positive association between education/literacy and obesity. Specialized health literacy campaigns should be designed to educate older adults who have a high education but lack of awareness. Also, this program should focus on combating sedentary behaviors and making informed nutritional choices in later life. Age-friendly environmental modification is also important. Local municipalities should develop safe physical environments—such as indoor activity spaces and well-lit walking paths—to encourage physical activity and mitigate sedentary lifestyles. Capacity building for caregivers and VHVs is also important. Local health authorities should empower caregivers and VHVs with specialized training on the double burden of malnutrition. This includes practical skills in preparing traditional Southern Thai dishes with reduced sugar and coconut milk, while maintaining optimal protein and micronutrient density.

Furthermore, future research such as an in-depth analysis of social support may be beneficial. Future mixed-methods or qualitative studies should explore caregiver intensity (frequency, duration, and quality of care) to better understand how distinct social support structures influence geriatric nutritional outcomes. Objective measurement methods are required in future trials, and researchers should utilize multi-day food records and body composition analyzers (e.g., bioelectrical impedance analysis) to minimize recall bias and objectively evaluate the distribution of skeletal muscle and adipose tissue.

## 5. Conclusions

In summary, nutritional status among older adults in Southern Thailand was associated with multiple demographic, socio-economic, behavioral, clinical, anthropometric, and community-level factors. Advanced age and continued, physically demanding work were linked to a higher likelihood of underweight, while female sex, higher education, literacy, dyslipidemia, and abdominal obesity were associated with increased risks of overweight and obesity. Regular, moderate physical activity was associated with lower risks of excess nutritional status, and WC was identified as an important complementary indicator to BMI for detecting abdominal obesity and cardiometabolic risk. These findings highlight the need for multi-level public health strategies that address both undernutrition and obesity through integrated BMI and WC screening, locally tailored nutritional intervention, physical activity promotion, chronic disease management, and strengthened community-level follow-up. In the Southern Thai context, effective nutritional policy should account for agricultural livelihoods, transitions in physical activity after older age, local food culture, caregiver availability, and the practical roles of VHVs and sub-district health systems.

## Figures and Tables

**Table 1:** Distribution and factors associated with BMI categories among Thai older adults.

Variables	Overall (N = 13,458)	Underweight (BMI < 18.5)	Normal Weight (BMI 18.5–22.9)	Overweight (BMI 23.0–24.9)	Obesity Class I (BMI 25.0–29.9)	Obesity Class II (BMI ≥ 30.0)	χ^2^	*p*-Value
	*n* (%)	*n* (%)	*n* (%)	*n* (%)	*n* (%)	*n* (%)		
Overall	13,458 (100.0)	1034 (7.7)	5654 (42.1)	2953 (21.9)	3005 (22.3)	812 (6.0)	—	—
1. Demographic and Socio-economic Factors								
Age Group							242.36	<0.001
60–69 years	6659 (49.5)	329 (4.9)	2692 (40.4)	1568 (23.5)	1629 (24.5)	441 (6.6)		
70–79 years	4168 (31.0)	374 (9.0)	1758 (42.2)	875 (21.0)	919 (22.0)	242 (5.8)		
≥80 years	2625 (19.5)	331 (12.6)	1205 (45.9)	510 (19.4)	456 (17.4)	123 (4.7)		
Sex							118.52	<0.001
Female	7981 (59.3)	591 (7.4)	3271 (41.0)	1625 (20.4)	1899 (23.8)	595 (7.5)		
Male	5471 (40.7)	443 (8.1)	2383 (43.6)	1328 (24.3)	1106 (20.2)	211 (3.9)		
Education Level							88.85	<0.001
No formal education	909 (6.8)	101 (11.1)	408 (44.9)	159 (17.5)	196 (21.6)	45 (5.0)		
Primary school	10,151 (75.4)	800 (7.9)	4301 (42.4)	2188 (21.6)	2229 (22.0)	633 (6.2)		
Secondary school	1707 (12.7)	101 (5.9)	715 (41.9)	418 (24.5)	378 (22.1)	95 (5.6)		
Bachelor’s degree or higher	681 (5.1)	32 (4.7)	227 (33.3)	187 (27.5)	202 (29.7)	33 (4.8)		
Literacy							240.32	<0.001
Literate	10,527 (78.2)	743 (7.1)	4325 (41.1)	2401 (22.8)	2420 (23.0)	638 (6.1)		
Semi-literate	583 (4.3)	55 (9.4)	246 (42.2)	101 (17.3)	135 (23.2)	46 (7.9)		
Illiterate	742 (5.5)	101 (13.6)	348 (46.9)	124 (16.7)	133 (17.9)	36 (4.9)		
Work Employed							110.07	<0.001
Yes	6660 (49.5)	637 (9.6)	2899 (43.5)	1316 (19.8)	1392 (20.9)	416 (6.2)		
No	6792 (50.5)	397 (5.8)	2755 (40.6)	1637 (24.1)	1613 (23.7)	390 (5.7)		
Income Adequacy							6.68	0.154
Yes	2287 (17.0)	192 (8.4)	946 (41.4)	470 (20.6)	530 (23.2)	149 (6.5)		
No	11,165 (83.0)	842 (7.5)	4708 (42.2)	2483 (22.2)	2475 (22.2)	657 (5.9)		
2. Health Behaviors								
Physical Activity Frequency							55.48	<0.001
Regular (>3 times/week)	6082 (45.2)	480 (7.9)	2508 (41.2)	1355 (22.3)	1343 (22.1)	396 (6.5)		
Moderate (3 times/week)	5650 (42.0)	385 (6.8)	2500 (44.2)	1249 (22.1)	1219 (21.6)	297 (5.3)		
Occasional (1–2 times/week)	25 (0.2)	4 (16.0)	13 (52.0)	5 (20.0)	2 (8.0)	1 (4.0)		
Never	1438 (10.7)	150 (10.4)	555 (38.6)	280 (19.5)	359 (25.0)	94 (6.5)		
Dietary Behaviors							16.90	0.031
Good	11,094 (82.4)	822 (7.4)	4679 (42.2)	2468 (22.2)	2461 (22.2)	664 (6.0)		
Moderate	531 (3.9)	46 (8.7)	207 (39.0)	109 (20.5)	125 (23.5)	44 (8.3)		
Poor	1618 (12.0)	151 (9.3)	676 (41.8)	329 (20.3)	369 (22.8)	93 (5.7)		
Engagement in Hobbies							23.24	<0.001
Yes	12,720 (94.5)	963 (7.6)	5372 (42.2)	2760 (21.7)	2853 (22.4)	772 (6.1)		
No	566 (4.2)	70 (12.4)	253 (44.7)	102 (18.0)	107 (18.9)	34 (6.0)		
Activities of Daily Living							25.75	<0.001
Independent	13,052 (97.0)	991 (7.6)	5490 (42.1)	2880 (22.1)	2918 (22.4)	773 (5.9)		
Semi-dependent	276 (2.1)	28 (10.1)	113 (40.9)	45 (16.3)	60 (21.7)	30 (10.9)		
Dependent	93 (0.7)	14 (15.1)	39 (41.9)	18 (19.4)	19 (20.4)	3 (3.2)		
Alcohol Consumption							93.45	<0.001
Never	9548 (71.0)	691 (7.2)	3891 (40.8)	2052 (21.5)	2240 (23.5)	674 (7.1)		
Former drinker	670 (5.0)	44 (6.6)	296 (44.2)	151 (22.5)	152 (22.7)	27 (4.0)		
Occasional drinker	1449 (10.8)	93 (6.4)	621 (42.9)	394 (27.2)	292 (20.2)	49 (3.4)		
Daily drinker	673 (5.0)	68 (10.1)	299 (44.4)	162 (24.1)	127 (18.9)	17 (2.5)		
Smoking Status							117.02	<0.001
Never smoked	9262 (68.8)	670 (7.2)	3764 (40.6)	1953 (21.1)	2204 (23.8)	671 (7.2)		
Former smoker	1985 (14.8)	157 (7.9)	843 (42.5)	484 (24.4)	429 (21.6)	72 (3.6)		
Current smoker	1647 (12.2)	150 (9.1)	745 (45.2)	409 (24.8)	293 (17.8)	50 (3.0)		
3. Clinical Factors								
Hypertension							25.79	<0.001
Yes	6786 (50.4)	446 (6.6)	2868 (42.3)	1497 (22.1)	1548 (22.8)	427 (6.3)		
No	6667 (49.6)	588 (8.8)	2787 (41.8)	1456 (21.8)	1457 (21.9)	379 (5.7)		
Diabetes Mellitus							42.64	<0.001
Yes	4425 (32.9)	260 (5.9)	1833 (41.4)	1065 (24.1)	1005 (22.7)	262 (5.9)		
No	9028 (67.1)	774 (8.6)	3822 (42.3)	1888 (20.9)	2000 (22.2)	544 (6.0)		
Dyslipidemia							37.76	<0.001
Yes	1501 (11.2)	109 (7.3)	579 (38.6)	312 (20.8)	363 (24.2)	138 (9.2)		
No	11,952 (88.8)	925 (7.7)	5076 (42.5)	2641 (22.1)	2642 (22.1)	668 (5.6)		
Asthma							18.11	0.001
Yes	240 (1.8)	34 (14.2)	107 (44.6)	43 (17.9)	42 (17.5)	14 (5.8)		
No	13,213 (98.2)	1000 (7.6)	5548 (42.0)	2910 (22.0)	2963 (22.4)	792 (6.0)		
Heart Disease							11.40	0.022
Yes	1270 (9.4)	102 (8.0)	581 (45.7)	240 (18.9)	271 (21.3)	76 (6.0)		
No	12,183 (90.6)	932 (7.7)	5074 (41.6)	2713 (22.3)	2734 (22.4)	730 (6.0)		
Knee Osteoarthritis							7.49	0.112
Yes	266 (2.0)	17 (6.4)	122 (45.9)	42 (15.8)	67 (25.2)	18 (6.8)		
No	13,187 (98.0)	1017 (7.7)	5533 (42.0)	2911 (22.1)	2938 (22.3)	788 (6.0)		
Thyroid Disease							6.15	0.188
Yes	144 (1.1)	8 (5.6)	51 (35.4)	33 (22.9)	39 (27.1)	13 (9.0)		
No	13,309 (98.9)	1026 (7.7)	5604 (42.1)	2920 (21.9)	2966 (22.3)	793 (6.0)		
Musculoskeletal Disorders							4.46	0.348
Yes	42 (0.3)	4 (9.5)	12 (28.6)	10 (23.8)	14 (33.3)	2 (4.8)		
No	13,411 (99.7)	1030 (7.7)	5643 (42.1)	2943 (21.9)	2991 (22.3)	804 (6.0)		
Abdominal Obesity							1137.63	<0.001
Yes	5446 (40.5)	224 (4.1)	1727(31.7.)	1207 (22.2)	1695 (31.1)	593 (10.9)		
No	8007 (59.5)	810 (10.1)	3960 (49.5)	1746 (21.8)	1278 (16.0)	213 (2.7)		
4. Social and Community Support								
Caregiver Support							20.71	<0.001
Yes	9116 (67.7)	652 (7.2)	3826 (42.0)	2041 (22.4)	2026 (22.2)	571 (6.3)		
No	3024 (22.5)	288 (9.5)	1261 (41.7)	624 (20.6)	677 (22.4)	174 (5.8)		
Village Health Volunteers							29.72	<0.001
Yes	12,047 (89.5)	957 (7.9)	5082 (42.2)	2596 (21.5)	2668 (22.1)	744 (6.2)		
No	652 (4.8)	26 (4.0)	257 (39.4)	183 (28.1)	157 (24.1)	29 (4.4)		
Healthcare Services (Local Gov/Health Center)							46.22	<0.001
Yes	7253 (53.9)	537 (7.4)	3064 (42.2)	1707 (23.5)	1588 (21.9)	357 (4.9)		
No	4620 (34.3)	351 (7.6)	1827 (39.5)	993 (21.5)	1104 (23.9)	345 (7.5)		
Health Promotion Activities							27.24	<0.001
Yes	8268 (61.4)	606 (7.3)	3460 (41.8)	1929 (23.3)	1824 (22.1)	449 (5.4)		
No	3703 (27.5)	297 (8.0)	1458 (39.4)	787 (21.3)	900 (24.3)	261 (7.0)		
Physician Services							2.42	0.659
Yes	10,852 (80.6)	828 (7.6)	4476 (41.2)	2450 (22.6)	2443 (22.5)	655 (6.0)		
No	1616 (12.0)	115 (7.1)	652 (40.3)	377 (23.3)	382 (23.6)	90 (5.6)		
Nursing Services							6.30	0.178
Yes	10,587 (78.7)	819 (7.7)	4349 (41.1)	2394 (22.6)	2392 (22.6)	633 (6.0)		
No	1248 (9.3)	94 (7.5)	494 (39.6)	262 (21.0)	317 (25.4)	81 (6.5)		
Annual Health Screening							6.32	0.176
Yes	9984 (74.2)	745 (7.5)	4115 (41.2)	2286 (22.9)	2243 (22.5)	595 (6.0)		
No	1713 (12.7)	147 (8.6)	720 (42.0)	352 (20.5)	389 (22.7)	105 (6.1)		

N = 13,458. Data are presented as *n* (%). BMI = body mass index. χ^2^ = chi-square statistic. BMI categories are based on Asian-specific cutoffs. *p*-values are derived from chi-square tests.

**Table 2:** Multinomial logistic regression table.

Variable	Category	BMI < 18.5 (Underweight)		BMI 23–24.9 (Overweight)		BMI 25.0–29.9 (Obesity Class I)		BMI ≥ 30.0 (Obesity Class II)	
		OR (95% CI)	*p*	OR (95% CI)	*p*	OR (95% CI)	*p*	OR (95% CI)	*p*
Age group	60–69	Reference	-	Reference	-	Reference	-	Reference	-
	70–79	1.53 (1.22–1.92)	<0.001	0.86 (0.75–0.99)	0.029	0.76 (0.66–0.87)	<0.001	0.76 (0.60–0.96)	0.020
	≥80	1.81 (1.41–2.33)	<0.001	0.79 (0.67–0.93)	0.006	0.55 (0.46–0.66)	<0.001	0.46 (0.34–0.63)	<0.001
Sex	Male	Reference	-	Reference	-	Reference	-	Reference	-
	Female	0.94 (0.73–1.20)	0.593	1.06 (0.90–1.24)	0.508	1.33 (1.13–1.56)	0.001	1.79 (1.34–2.38)	<0.001
Education level	No formal education	Reference	-	Reference	-	Reference	-	Reference	-
	Primary	1.11 (0.73–1.70)	0.632	1.12 (0.81–1.53)	0.496	0.80 (0.59–1.09)	0.163	1.59 (0.92–2.74)	0.095
	Secondary	0.95 (0.58–1.58)	0.854	1.04 (0.73–1.49)	0.831	0.77 (0.54–1.09)	0.135	1.79 (0.98–3.29)	0.060
	Graduate	1.15 (0.60–2.18)	0.674	1.62 (1.08–2.44)	0.020	1.33 (0.89–1.97)	0.164	1.19 (0.56–2.51)	0.656
Literacy	Illiterate	Reference	-	Reference	-	Reference	-	Reference	-
	Semi-literate	0.63 (0.37–1.09)	0.097	0.93 (0.62–1.40)	0.728	1.49 (0.98–2.28)	0.065	1.32 (0.68–2.59)	0.413
	Literate	0.67 (0.43–1.04)	0.075	1.02 (0.72–1.45)	0.900	1.64 (1.13–2.37)	0.009	0.98 (0.54–1.79)	0.955
Employment status	Unemployed	Reference	-	Reference	-	Reference	–	Reference	-
	Employed	1.43 (1.17–1.76)	0.001	0.87 (0.76–0.99)	0.030	0.86 (0.76–0.98)	0.028	1.01 (0.82–1.26)	0.906
Consumption behaviors	Bad	Reference	-	Reference	-	Reference	-	Reference	-
	Moderate	0.73 (0.40–1.32)	0.295	0.88 (0.59–1.31)	0.529	1.38 (0.93–2.07)	0.114	1.30 (0.70–2.42)	0.405
	Good	0.79 (0.53–1.17)	0.241	1.04 (0.79–1.36)	0.798	1.30 (0.97–1.74)	0.085	1.13 (0.71–1.79)	0.601
Exercise	Never	Reference	-	Reference	-	Reference	-	Reference	-
	Mild	1.68 (0.39–7.30)	0.492	~0	-	~0	-	~0	-
	Moderate	0.77 (0.49–1.21)	0.265	0.72 (0.53–0.97)	0.031	0.55 (0.41–0.73)	<0.001	0.39 (0.26–0.60)	<0.001
	Good	0.80 (0.51–1.25)	0.332	0.94 (0.70–1.27)	0.677	0.73 (0.55–0.97)	0.028	0.62 (0.41–0.94)	0.024
ADL	Dependent	Reference	-	Reference	-	Reference	-	Reference	-
	Semi-dependent	0.59 (0.23–1.56)	0.288	0.69 (0.29–1.66)	0.412	0.94 (0.40–2.20)	0.888	4.55 (0.54–38.54)	0.164
	Independent	0.64 (0.29–1.42)	0.274	1.21 (0.58–2.53)	0.604	1.11 (0.53–2.31)	0.781	3.92 (0.50–30.47)	0.192
Hobbies	Yes	1.77 (0.78–3.99)	0.170	0.89 (0.57–1.40)	0.620	0.80 (0.51–1.24)	0.321	1.72 (0.70–4.19)	0.237
HT	Yes	0.85 (0.69–1.05)	0.137	1.01 (0.88–1.16)	0.888	1.17 (1.02–1.34)	0.028	0.94 (0.75–1.18)	0.591
DM	Yes	0.74 (0.58–0.93)	0.009	1.15 (1.01–1.32)	0.042	0.88 (0.76–1.02)	0.079	0.89 (0.70–1.13)	0.332
CHOL	Yes	1.28 (0.96–1.72)	0.092	1.15 (0.95–1.40)	0.145	1.29 (1.07–1.55)	0.007	2.04 (1.56–2.68)	<0.001
HD	Yes	0.82 (0.56–1.20)	0.306	0.65 (0.50–0.84)	0.001	0.72 (0.55–0.94)	0.015	1.04 (0.69–1.57)	0.843
Asthma	Yes	1.76 (1.07–2.89)	0.026	0.70 (0.44–1.12)	0.699	0.65 (0.40–1.05)	0.076	0.85 (0.41–1.74)	0.647
Abd obesity	Yes	0.63 (0.36–1.11)	0.114	1.90 (1.46–2.48)	<0.001	3.39 (2.63–4.39)	<0.001	6.01 (3.98–9.06)	<0.001
Alcohol consumption	Never	Reference	-	Reference	-	Reference	-	Reference	-
	Sometimes	0.68 (0.45–1.02)	0.062	1.29 (1.01–1.63)	0.039	1.21 (0.93–1.57)	0.158	1.02 (0.62–1.70)	0.926
	Daily	1.36 (0.87–2.12)	0.177	1.14 (0.84–1.54)	0.415	1.01 (0.72–1.41)	0.974	0.78 (0.39–1.60)	0.504
	Former drinker	0.66 (0.40–1.08)	0.095	0.90 (0.66–1.23)	0.504	1.16 (0.84–1.59)	0.372	0.86 (0.45–1.62)	0.637
Smoking	Never	Reference	-	Reference	-	Reference	-	Reference	-
	Current	1.43 (0.98–2.07)	0.061	1.08 (0.85–1.38)	0.509	0.77 (0.59–1.01)	0.056	0.60 (0.36–1.01)	0.055
	Former	1.22 (0.82–1.82)	0.317	1.12 (0.87–1.44)	0.382	0.89 (0.68–1.16)	0.397	0.69 (0.41–1.15)	0.154
Caregiver	Yes	0.87 (0.70–1.09)	0.224	1.02 (0.88–1.18)	0.818	0.91 (0.78–1.05)	0.199	0.93 (0.73–1.19)	0.558
Health volunteer	Yes	1.21 (0.72–2.03)	0.481	1.28 (0.92–1.79)	0.148	1.07 (0.76–1.50)	0.693	0.87 (0.47–1.60)	0.648
Health service SOA	Yes	0.99 (0.78–1.27)	0.954	0.99 (0.85–1.15)	0.865	0.97 (0.83–1.13)	0.687	0.79 (0.61–1.02)	0.065
Health promotion participation	Yes	0.80 (0.62–1.03)	0.081	1.10 (0.93–1.29)	0.255	0.91 (0.78–1.07)	0.253	0.88 (0.68–1.14)	0.331

## Data Availability

The data supporting the findings of this study are available from the corresponding author upon reasonable request. The data are not publicly available due to privacy and ethical restrictions related to participant information.

## Abbreviations

The following abbreviations are used in this manuscript:

AOR	Adjusted odds ratio
BMI	Body mass index
CI	Confidence interval
SDH	Social determinants of health
VHV	Village health volunteer
WC	Waist circumference
